# Adapting Motor Imagery Training Protocols to Surgical Education: A Systematic Review and Meta-Analysis

**DOI:** 10.1177/1553350621990480

**Published:** 2021-03-12

**Authors:** Mary S. L. Goble, Nicholas Raison, Ayah Mekhaimar, Prokar Dasgupta, Kamran Ahmed

**Affiliations:** 1 MRC Centre for Transplantation, Guy’s Hospital, 4616 King’s College London, UK; 2 Department of Urology, 4616 Guy’s and St Thomas’ NHS Foundation Trust, King’s Health Partners, London, UK

**Keywords:** motor imagery, mental training, medical education, surgical education, curricula

## Abstract

*Objective.* Motor imagery (MI) is widely used to improve technical skills in sports and has been proven to be effective in neurorehabilitation and surgical education. This review aims to identify the key characteristics of MI protocols for implementation into surgical curricula. *Design.* This study is a systematic review and meta-analysis. PubMed, MEDLINE, Embase and PsycINFO databases were systematically searched. The primary outcome was the impact of MI training on measured outcomes, and secondary outcomes were study population, MI intervention characteristics, study primary outcome measure and subject rating of MI ability (systematic review registration: PROSPERO CRD42019121895). *Results.* 456 records were screened, 60 full texts randomising 2251 participants were reviewed and 39 studies were included in meta-analysis. MI was associated with improved outcome in 35/60 studies, and pooled analysis also showed improved outcome on all studies with a standardised mean difference of .39 (95% CI: .12, .67, *P* = .005). In studies where MI groups showed improved outcomes, the median duration of training was 24 days (mode 42 days), and the median duration of each individual MI session was 30 minutes (range <1 minute-120 minutes). *Conclusions.* MI training protocols for use in surgical education could have the following characteristics: MI training delivered in parallel to existing surgical training, in a flexible format; inclusion of a brief period of relaxation, followed by several sets of repetitions of MI and a refocusing period. This is a step towards the development of a surgical MI training programme, as a low-cost, low-risk tool to enhance practical skills.

## Introduction

Surgical education has been increasingly reliant on training methods which involve simulation, ranging from simple bench models to virtual reality simulation and box trainers.^1^ Motor imagery (MI) can be described as a form of simulation; it consists of imagining oneself performing a voluntary movement, without physically moving.^2^ It has also been called mental practice (MP), mental training and mental imagery.

Motor imagery has been proven to be effective at improving technical skills in various fields,^3-6^ and structured training programmes which incorporate this concept are reported in the literature. In sports psychology, MI has been integrated in several models such as the PETTLEP model^7^ which delivers a format of training applicable to different sports. In the field of neurorehabilitation, Braun’s review^8^ identified the elements which correlate with effective training outcomes.

Several studies have successfully shown that this method can also be adapted to surgical training^9-11^: Immenroth et al^11^ used MI for training in laparoscopy cholecystectomy via one-on-one mental training sessions, where trainees memorised the operation primer and visualised their inner perception of the operation based on this. Louridas et al^12^ developed and tested a script based on MI to perform laparoscopic jejunojejunostomy, using visual and kinaesthetic (tactile) cues. Despite encouraging results, these studies allow limited application for MI training outside of the specific surgical procedures they were designed for.

The aforementioned areas of neurorehabilitation, sport psychology and training in specific surgical procedures use common principles of MI to achieve motor improvement. Current understanding of the neurological mechanism of MI is dominated in the literature by Jeannerod’s central motor theory.^7,13-16^ It underpins the hypothesis that a degree of functional equivalence exists between MI, and motor preparation and execution, and that they share common neural substrate.^7,13,14^ Empirical evidence supporting the functional equivalence concept can be seen at different levels of control, namely central (in the frontal and parietal lobes^17^), peripheral (via increased heart rate and respiratory rate^18^) and behavioural (via mental chronometry^18-20^). This mechanism is applied to any motor development using MI, regardless of the type of skill being targeted. Based on this understanding, a cross-disciplinary use of MI protocols can be explored in order to identify important elements of MI training.

Protocols incorporating MI in medical education are not readily available in the literature,^10^ and there has so far been no rigorous approach regarding the evaluation of the format in which MI training should be incorporated into surgical education programmes.

The aim of this review and meta-analysis is to identify the components necessary to a training protocol for surgical education which uses MI. This will be done by gathering evidence from fields which have successfully used this method for decades.^21^ The primary outcome will be the effectiveness of a protocol using MI training, measured through different outcome measures due to the diversity of studies included. The secondary outcomes will be protocol components.

This review will be structured according to the PRISMA checklist for systematic reviews and meta-analyses.

## Methods

### Protocol and Registration

The review has been registered on PROSPERO (registration number: CRD42019121895).

### Eligibility Criteria

In order to limit this review to evidence of the highest standard of quality, only randomised controlled trials (RCTs) of the use of MI in any discipline were used. Inclusion criteria were as follows: RCTs published up until December 2018; studies in English, French and Spanish only; studies of MI training programmes which measured an objective outcome for a specific voluntary skill; studies which included a protocol based on imagining a movement. Exclusion criteria were as follows: studies which were not RCTs; studies in which MI training was combined with simulation training; studies in which MI training was done in conjunction with functional magnetic resonance imaging (fMRI), electroencephalograms (EEG), electromyography (EMG), transcutaneous electric nerve stimulation (TENS), electroacupuncture or hypnosis. However, studies using fMRI, EEG or EMG only as part of pre- and post-intervention evaluation, and not during the intervention period (as may be the case in bio-neurofeedback), were included, providing they met the other eligibility criteria.

### Search Strategy and Study Selection

The following databases were searched from inception by 2 authors: PubMed, MEDLINE, Embase and PsycINFO. The following combination of index terms was used: ‘randomised controlled trial’, ‘RCT’, ‘mental imagery’, ‘MP’, ‘mental training’ and ‘MI’. Detail of the search strategy is presented in Appendix A. There were no registered MeSH terms pertaining to this topic. The last date of search was January 12, 2019. Titles of studies were screened for selection. The abstracts were read. Where necessary, the full text was read. At each step, the studies were assessed according to the exclusion and inclusion criteria. Studies selected for inclusion were uploaded onto RefWorks and checked for duplication. Both authors completed the search independently and compared results, incongruities were resolved by discussion. (See Appendix B for list of abbreviations used in Tables 1 and 2).

**Table 1.** Main Study Characteristics.

Study	MI Ability Measurement	N° Participants	Population Type	Interventions	Duration of MP Session (minutes) × Total Amount of Sessions × Intervention Duration (days)	Primary Outcome Measures	Intervention Group Compared to Control
Abraham 2018	MIQ-RS, KVIQ-20 VMIQ-2	20	Parkinson’s disease	MP vs control	120 min × 10 × 14 days	TUGT, 6-min walk test, FGS, 30 sec chair stand, 360° turn test and PRT	Improved
Asa 2014	No	36	Healthy	MP only vs PP only vs control	25 min × 1 × 1 days	Speed and accuracy of task performance	No difference
Bathalon 2005	No	44	Healthy	MI and KG vs KG alone vs control	n/a × n/a × 14 days	OSCE	Improved
Bovend'Eerdt 2008	IST	11	Arm spasticity	PP vs MP and PP vs control	1.5 min × 32 × 56 days	Resistance to passive movement	No difference
Braun 2011	VMIQ-2	47	Parkinson’s disease	PP vs MP and PP vs control	20 min × 6 × 42 days	Walking performance using visual analogue scale	No difference
Braun 2012	No	36	Stroke patients	PP vs MP and PP vs control	n/a x 10 × 42 days	Numeric rating scales	No difference
Callow 2017	VMIQ-2	56	Healthy	IVI vs IVI and KIN vs control	2 min × 1 × 1 days	Time	IVI and KIN improved
Cho 2013	No	28	Stroke patients	MP vs control	15 min × 18 × 42 days	FRT, TUGT, 10- m walk test and FMA	Improved
Coker 2015	VMIQ-2	24	Healthy	VI vs KIN vs control	60 min (MP+PP) × 1 × 1 day	Hip movements recorded on motion capture system	No difference
Conlin 2016	MIQ-R	12	Healthy	MP vs control	n/a × 1 × 1 days	TSE and GE of mastoidectomy	No difference
Cunha 2017	MIQ-RS	15	Transtibial amputees	MP vs control	40 min × 12 × 28 days	Ground reaction forces	Improved
Dilek 2018	No	36	Distal radius fracture	MP vs control	100 min × 1680 × 56 days	Pain, wrist and forearm active ROM, grip strength, DASH questionnaire and MHQ	Improved
Eldred-Evans 2013	No	64	Healthy	VRS vs standard training and MP vs VRS training and MP vs control	30 min × 3 × 6 days	Time, precision, accuracy and overall performance on BT and VRS	MP groups improved
Frenkel 2014	No	18	Healthy	MP vs control	15 min × 315 × 21 days	ROM	Improved
Geoffrion 2012	No	79	Healthy	MP vs control	n/a × 1 × 1 days	GRS	No difference
Gomes 2014	No	60	Healthy	MP vs PP vs MP and then PP vs PP and then MP vs control	.1 min × 24 × 1 days	Time	PP, MP and PP and PP and MP improved
Guillot 2009	Mental chronometry	14	Burn patients	MP vs control	n/a × 10 × 14 days	Goniometric data	Improved
Hemayattalab 2009	No	40	Intellectually disabled patients	MP vs PP vs MP and then PP vs PP and then MP vs control	30 min × 24 × 24 days	Free throw test	PP improved most
Hidalgo-Perez 2015	No	40	Healthy	MCTE and MI vs control	15 min × 20 × 30 days	Craniocervical neuromotor control, joint position error and fatigue after effort	Improved
Hosseini 2012	MMSE, VMIQ and VVIQ	30	Healthy	OT and MP vs control	15 min × 15 × 35 days	TUGT	Improved
Hoyek 2014	MIQ-R	16	Shoulder impingement	MP and PP vs control	15 min × 9 × 21 days	Shoulder function, ROM and pain	Improved
Ietswaart 2011	Task chronometry	121	Stroke patients	MP and standard rehabilitation vs standard rehabilitation and non-motor mental rehearsal vs control	39 min × 20 × 28 days	ARAT	No difference
Immenroth 2007	No	98	Healthy	MP and PP vs PP only vs control	60 min × 2 × 2 days	OSATS	No difference
Jungmann 2011	Mental chronometry	40	Healthy	MP and VR training vs control	3 min × 4 × 4 days	Time, tip trajectory and instrument collision	No difference
Kim 2013	VMIQ-2	30	Stroke patients	Standard therapy and AO vs standard therapy and MP vs control	20 min × 20 × 28 days	TUGT, FRT, WAQ and FAC	Improvement in AO group
Kim 2018	VMIQ	16	Stroke patients	CMIT and MP vs control	10 min × 10 × 14 days	Motor evoked potential amplitude, 3-D motion analysis, JT test and motor activity log	No difference
Komesu 2009	No	68	Healthy	MP vs control	20 min × 1 × 1 day	GSOP	No difference
Lebon 2011	No	12	Torn ACL patients	Standard physiotherapy and MP vs control	15 min × 180 × 31 days	Muscle activation	Improved
Lim 2016	No	20	Healthy	MP and PP vs and control	60 min × 1 × 1 day	Technical achievement	No difference
Liu 2004	No	46	Stroke patients	MP and standard rehabilitation vs control	60 min × 15 × 21 days	Performance on 15 tasks, FMA and CTT	No difference
Liu 2009	No	34	Stroke patients	MP and conventional therapy vs control	60 min × 15 × 21 days	Performance on 15 tasks	No difference
Liu 2009	No	35	Post-stroke patients	MP and conventional therapy vs control	60 min × 15 × 21 days	Evaluation of skills	No difference
Losana-Ferrer 2018	MIQ	60	Healthy	MP vs AO vs control	6 min × n/a × 10 days	Hand grip strength, EMG and IM oxygenation	Improved (not on IM oxygenation)
Louridas 2015	MIQ and MIQ-R	20	Healthy	PP and MP vs control	n/a × n/a × 7 days	OSATS and bariatric OSATS	No difference
Malouin 2009	KVIQ	12	Stroke patients	MP and PP vs cognitive training and PP vs control	n/a × 12 × 28 days	Limb loading	Improved
Maring 1990	No	26	Healthy	MP and PP vs control	n/a x 1 x 1 days	Upper limb muscle activity	Improved
Mendoza 1978	No	32	Healthy	MP only vs MP with simulated movement vs PP only vs control	15 min × 6 × 6 days	Success in dart throwing	PP improved most
Millard 2001	No	60	Healthy	MP vs PP vs MP and PP vs control	30 min × 3 × 3 days	Wet exit attempts scored	MP and PP improved most
Mulla 2012	No	41	Healthy	BT vs BT and additional practice vs VRS training vs MP vs control	15 min × 7 × 7 days	Assessed on BT and VRS on time, precision, accuracy and performance	MP performed worst apart from control
Nicholson 2018	KVIQ	30	Healthy	MP only vs PP only vs control	25 min × 1 × 1 day	Gait speed, gait variability using GAITRite and timed up and go	No difference
Nilsen 2012	VMIQ-2	19	Stroke patients	PP and internal MP vs PP and external MP vs control	18 min × 12 × 42 days	FM and JT test	Improved
Oostra 2015	MIQ-RS	44	Stroke patients	Standard rehabilitation and MP vs control	30 min × 30 × 42 days	10-m walk test and FM	No difference
Page 2005	No	11	Stroke patients	PP and MP vs PP only vs control	30 min x 12 × 42 days	Motor activity log and ARAT	Improved
Page 2007	No	32	Stroke patients	MP and standard rehabilitation vs control	30 min × 12 × 42 days	ARAT and FM	Improved
Page 2009	No	10	Stroke patients	mCIT and MP vs control	30 min × 30 × 70 days	ARAT and FM	Improved
Page, S. 2011	No	29	Stroke patients	MP vs MP vs MP (different lengths) vs control	40 min × 30 × 70 days	FM and ARAT	Improved
Park 2015	VMIQ	30	Stroke patients	MP and standard rehabilitation vs control	10 min x 20 × 28 days	LBT and SCT	Improved
Sanders 2004	No	65	Healthy	2 sessions PP and 1 MP vs 1 PP and 2 MP vs control	30 min × 3 × 21 days	GRS	No difference
Sanders 2008	Revised Minnesota test	64	Healthy	MP vs control	30 min × 2 × 24 days	Surgical behaviour	Improved
Santiago 2015	MIQ-R	20	Parkinson’s disease	MP and standard therapy vs control	n/a × 1 × 1 day	Gait analysis	No difference
Schuster 2012	KVIQ	39	Stroke patients	MP added to physio vs MP embedded into physio vs control	45 min × 6 × 14 days	Time taken to perform skill	No difference
Seebacher 2017	No	112	MS patients	Music and MI vs metronome MI vs control	17 min × 24 × 28 days	Walking speed	Improved
Sharp 2014	No	18	Spinal cord injury patients	MP with overground training vs control	30 min × 24 × 56 days	Gait velocity	No difference
Sidaway 2005	No	24	Healthy	MP vs PP vs control	15 min × 12 × 28 days	Isometric torque and percentage improvement	No difference
Stenekes 2009	VMIQ	28	Post flexor tendon repair	MP and conventional therapy vs control	.6 min × 336 × 42 days	Preparation time of finger flexion	Improved
Timmermans 2013	VMIQ	42	Stroke patients	Standard therapy and MP vs control	10 min × 126 × 42 days	FM, FAT, WMFT and accelerometry	No difference and improved on FAT
Vergeer 2006	VVIQ	47	Healthy	MI vs SI vs control	30 min × 11 × 28 days	Flexibility and comfort	Improved in comfort and no difference in flexibility
Wilson 2002	No	54	Motor coordination problems	MI vs PP vs control	60 min × 5 × 35 days	MABC	PP improved most
Wilson 2016	No	36	Motor coordination problems	MI vs PP	60 min × 5 × 35 days	MABC	No difference

**Table 2.** Protocol Components for MI Training.

Study	MP Protocol Detail	Control Group
Abraham 2018	16 h training. 5x 2 h sessions every week for 2 weeks. Delivered in group by therapist. First session: Introduction to imagery. Subsequent sessions: 15 min warm-up, 35 min practice, 35 min practice, 20 min movement session and 5 min cool down. Overall structure: Acquire imagery skills and technique, understand anatomy and function and use imagery for improvement	Standard training and in-home learning and exercise programme following the same pattern as the intervention group
Asa 2014	MP instructions emphasised kinaesthetic imagery, keeping eyes closed	No training
Bathalon 2005	MI and KG group: KG teaching broke down task into 8 steps, students performed task and 5 min teaching of mental imagery. Instructed to perform MI in their own time as often as possible	Standard ATLS training
Bovend'Eerdt 2008	Closed eyes, imagined limb in mind’s eye and imagined movement in mind’s eye. Performed the skill (stretch) physically whilst imagining it. Stretches held for 10-30 secs, 3 repetitions/stretch. MP done immediately prior to PP	PP and relaxation following the same pattern as the intervention group
Braun 2011	MP with therapist, then unguided. 1 log/week completed by participants to record MP behaviour. 6 weeks standard physiotherapy, 1 h/week in groups or 30 min 2x/week individually, of which MP for 20 min in groups or 10 min individually	Standard physiotherapy and relaxation following the same pattern as the intervention group
Braun 2012	6 weeks rehabilitation, at least 10 sessions of MP (conditional) and practice outside supervised therapy time (optional). 4-step programme: Explain concept, develop imagery technique, apply mental practice and consolidate	Regular rehabilitation + homework practising difficult tasks
Callow 2017	IVI script: First-person visual perspective. IVI and KIN script: First-person visual perspective and physical feelings	Participants answered arithmetic questions
Cho 2013	15 min MP: Videos of normal movement shown, explanation of movement by researcher and imagining normal movement based on visual material using kinaesthetic and visual imagery. 5 min relaxation. 30 min gait training 45 min/day, 3x/week for 6 weeks	Standard physiotherapy only
Coker 2015	Training block of x10 trials of skill to generate feedback. Then, practice sessions alternating PP (x5 repetitions) and MP (visual or kinaesthetic imagery, x20 repetitions of the task). Total blocks had x15 repetitions PP and x60 repetitions MP. Relaxation done before training	Mental arithmetic task
Conlin 2016	Relaxation, script read out loud and given in written format. Script based on transcript of audio recordings of 3 experts having identified steps in the procedure and reported visual, cognitive and kinaesthetic cues involved. Participants to actively imagine performing skill. Given copy of script to take home and review	Self-directed textbook study
Cunha 2017	40 min sessions, 3x/week for 4 weeks. First-person perspective and tasks of increasing difficulty. 10 tasks imagined in each session, then described	Standard training and non-motor task MP
Dilek 2018	Graded motor imagery. 3 stages. 1: 3 weeks of lateralisation: Identifying correct right and left hands from pictures, x3 each hour every day. 2: 3 weeks of MI, visualise own hand moving to posture in image shown, without physically moving, x3 each hour every day. 3: 2 weeks mirror therapy: Move own hand to posture in image shown, x10 every hour every day. All participants instructed to perform home exercise programme	Standard rehabilitation
Eldred-Evans 2013	Based on the Mackay nodal model of mental practice. Relaxation, guided visualisation of nodal points	Standard box training and additional self-practice
Frenkel 2014	Mental gait training procedure: (1) movement explained; (2) describe movement by observing it performed, practicing it on non-tested hand and concentrating on kinaesthetic properties; (3) break down into nodal points and connect points with kinaesthetic perception; (4) practice on non-tested hand with open eyes and closed eyes, perform visual imagery and kinaesthetic imagery and (5) practice on non-tested hand, perform kinaesthetic imagery of task and of an unrelated task. Completed a dairy to record completion of training. 1 × 60 min and then 3 × 30 min guided sessions. Followed by 15 min/day self-guided imagery	No training
Geoffrion 2012	MI script enumerated steps from textbook and added visual, cognitive and kinaesthetic performance details. Participants performed MP one-on-one with educator, then individually	Normal surgical training and encouraged to read textbook on skill
Gomes 2014	Instructions to use internal kinaesthetic perspective. Participants closed eyes, signal start of imagining and signal end of imagining	No training
Guillot 2009	Script detailing instructions of 2 motor tasks, encouraging self-representation of movements, sensory and kinaesthetic cues, staying immobile. Patients to perform MI during training sessions only. Regularly asked to describe nature of images after MI. Total 2 weeks, 5 MP sessions	Standard rehabilitation and neutral activities following the same pattern as the intervention group
Hemayattalab 2009	Using internal kinaesthetic imagery. PP: 30 repetitions of the skill/session. MP: 30 repetitions of imagining skill. MP and PP: MP for 12 sessions and then PP for 12 sessions. PP and MP: PP for 12 sessions and then MP for 12 sessions	No training
Hidalgo-Perez 2015	MI done just after PP. 1/day, 5 days/week, 30 days. 4 phases, 1 phase/week of intervention: 1- kinaesthetic imagery, 2- visual imagery, 3- movement observation therapy plus MI and 4- exercise execution with mirror feedback. Weekly email and phone reminders	MCTE only
Hosseini 2012	15 MP, then 30 min occupational therapy. MP: 5 min relaxation, 10 lying supine with eyes closed, asked to imagine skill in first person	Occupational therapy only, for 45 min
Hoyek 2014	4 movements imagined using internal visual imagery and kinaesthetic imagery. All movements shown before MP. Participants told to imagine movement as slowly and vividly as possible. Imagery script read to them. Each movement imagined 10 times, 5 sets of 2 separated by 30-s rest. 10 long sessions of physical therapy, 3x/week. MP exercises done during therapy sessions in rest times. 45 min physical therapy and 15 min MP.	Physical therapy training with neutral activities during rest time
Ietswaart 2011	12 x 45 min sessions with therapist 3 days/week: 30 min MP actively imagining basic movements, 10 min MP using videos and mirrors and 5 min covert MP, for example mentally rotating visual depiction of hands. 8 × 30 min sessions alone, 2 days/week: Audio tape instructing movements to be imagined. Patients to keep a log book. Total 4 weeks	Standard physiotherapy only
Immenroth 2007	Day 1: One-on-one mental training for 90 min. 30 min to learn primer by heart, recall wording of primer by external self-talk, relaxation exercise, visualisation in first person under supervision and then alone. Day 2: 30 min session repeating external self-talk and ideomotoric training under supervision	No intervention
Jungmann 2011	Completed 2 sessions VR training. Then, received CD-ROM with demonstration video of skill, checklist for skill steps and instructions on how to perform MP. Practised MP independently before second VR training session	VR training only
Kim 2013	5x/week, 30 min sessions, over 4 weeks: 20 min audio instructions and 10 min PP.	Standard therapy only
Kim 2018	Modified constraint-induced movement (CMIT) therapy for 1h and then MP for 10 min. Listened to audio while watching first-person perspective video for 4 min. Close eyes, relaxation for 2 min. Repeat audio only without video for 4 min. Audio included kinaesthetic mental practice. 5 days/week for 2 weeks	CMIT and listened to piano music for 10min
Komesu 2009	Perform MP 24-48 h before assessment. Imagine performing skill and describe to educator in detail	Standard surgical training and textbook study following the same pattern as the intervention group
Lebon 2011	Sat with legs extended. Relaxation done in initial few sessions only. Perceive muscle contractions and joint tension while imagining movement. 3 blocks of 10 imagined movement, 10 sec rest between imagined movements and 2 min rest between blocks. MP: 28-34 day programme. 12 × 15 min sessions, one every 2 days. Physiotherapy: 30 min every 2 days	Standard physiotherapy and neutral task following the same pattern as the intervention group
Lim 2016	60 min scripted mental imagery group training. After 20 min, independent mental rehearsal. After session, performed skill x3	Low-fidelity simulation training only
Liu 2004	Increasing difficulty of tasks. First week: Analyse task sequences with pictures and movies. Second week: Identify own problems. Third week: Imagine task being performed by self, physically perform task and videotape, view videotape and adjust problems. Repeat identification of problems and third week steps until proper method is achieved. 15 sessions MP, 1 h/day for 3 weeks and standard physiotherapy, 1 h/day for 5 days/week at a different time of day	Standard rehabilitation and neutral activities following the same pattern as the intervention group
Liu 2009	MP and conventional therapy, learning tasks of increasing difficulty. 1 h physical therapy and 1 h MP 5x/week for 3 weeks	Followed the same pattern of therapy as the intervention group with occupational therapy instead of MP
Liu 2009	Chunking-regulation-rehearsal strategy: Truncate task, self-reflect on abilities, feedback using video playback, mentally rehearsing and physically practising. MP and conventional therapy, learning tasks of increasing difficulty. 1 h physical therapy and 1 h MP 5x/week for 3 weeks	Physical practice and functional rehabilitation following the same pattern as the intervention group
Losana-Ferrer 2018	Sit on chair, imagine 10 physical repetitions for 3 sec each and 20 sec rest in between. 2 min break. Repeat imagery whilst also performing skill physically. 10 training days, 1st and 4th supervised and remainder at home. All groups told to practice at home and weekly reminders	Physical practice following the same pattern as intervention groups
Louridas 2015	Didactic lecture. In-person instructions on MP. Relaxation exercise. MP guided by MP script developed by interviewing experts and detailing visual and kinaesthetic cues, including common pitfalls in performance. Given script and videos of didactic teaching, 7 days to perform MP at home, with follow-up calls and feedback	Standard physical practice only following the same pattern as the intervention group
Malouin 2009	Approx 1 h training done in quiet room by physical therapist. MP done in blocks following one attempt of PP. Briefing on first-person imagery with focus on kinaesthetic imagery (sensory). Close eyes, imagine task. Number of mental repetitions increased with time. Live feedback on performance was given for first few sessions, via outcome measurement tool. 3/week for 4 weeks	No training
Maring 1990	Maximum voluntary contraction of muscle, 2 min visualisation with visual and kinaesthetic cues and no physical movement, physical practice of skill x10. Repeated x5	PP only and task demanding mental attention following the same pattern as the mental intervention group
Mendoza 1978	MP only: Sit with eyes closed, imagine performing skill whilst being aware of all sensory input, correcting for imagined misses. 2 × 15 min sessions/day for 6 days	No practice
Millard 2001	MP group: Watched video, taught mental practice and watched video + made entry in diary after each MP session. PP group: Watched demonstration, then did drill 3x/day for 3 days. PP and MP groups did both training	No training
Mulla 2012	25 min one-to-one mental training. Description and memorisation of motor skills involved, relaxation and internal and external visualisation of skills to perform. Student to practise at home 15 min/day every day	No training
Nicholson 2018	Sat in chair. Imagined completing obstacle course in first-person perspective. MP group: 20 imagined repetitions of a task. PP group: 20 physical repetitions of a task. In both: 30 sec rest between each trial and 5 minute rest after every 10 repetitions	25 min playing mentally stimulating games on iPad
Nilsen 2012	Listened to audio script of MP with visual and kinaesthetic detail. 2 min introduction instructing internal perspective (group 1) or external perspective (group 2). 5 min relaxation. 8 min focussed imagery with key components of task repeated several times. 3 min refocusing	Occupational therapy and relaxation following the same pattern as the intervention groups
Oostra 2015	30 min sessions, in quiet room with 2 therapists, sit down and eyes closed. 2 min relaxation, perform practice from internal perspective, with a visual and kinaesthetic mode. Content of sessions was familiarisation in week 1, specific gait problems week 2 and symmetry and velocity weeks 3 and 4	Standard rehabilitation and generic relaxation sessions following the same pattern as the intervention group
Page 2005	MP corresponded to focus therapy, which changed weekly. Audio tape: 5 min relaxation, then suggestions for internal, cognitive polysensory images, then 3-5 min refocusing. 30 min occupational therapy (PP) sessions 2 days/week for 6 weeks followed by 30 min MP	Occupational therapy and relaxation techniques
Page 2007	MP sessions directly after PP. Audio tape. 30 min total: 5 min relaxation, approx. 20 min suggestions for internal, cognitive polysensory images of skill performed in PP on the same day (several trials of imaging) and refocusing. Patients instructed not to do additional MP at home	Standard rehabilitation and relaxation following the same pattern as the intervention group
Page 2009	Audio tapes read by male psychologist delivered in quiet room. 5 min guided relaxation, 15-20 min motor imagery in first person using polysensory cues and 5 min refocusing. Instructed to not do self-directed practice. 3 days/week for 10 weeks	mCIT only
Page, S. 2011	Audiotaped MP intervention listened to in private room. 5 min relaxation (imagine themselves in nice place and contract/relax muscles), followed by suggestions for sensory images related to use of the arm and finishing with 5 min refocusing into the room. Opening and closing 5 minutes held constant in varying lengths of MP practice. Group 1: MP for 20 minutes, group 2: MP for 40 min and group 3: 60 min	Same baseline rehabilitation sessions and audiotaped sham intervention directly after the rehabilitation session
Park 2015	Sit with eyes closed, imagine scene while listening to voice of instructor for 10 min and give verbal feedback. 10 repetitions of each skill. Break in between skills for relaxation and internal concentration. 5 days/week for 4 weeks	Standard rehabilitation only
Sanders 2004	Relaxation by psychologist, then verbal imagery instruction in making incision, suturing and knot tying by physician while visualising. 30 min long sessions, 1/week	3 sessions PP only
Sanders, W. 2008	Relaxation by psychologist, then guided imagery instruction in making incision and performing sutures. 30 min	Same baseline training and 2 additional sessions of reading. Same instructional time as the intervention group
Santiago 2015	1. Identified problems in gait. 2. Memorised phases of normal gait with images, performed gait 5x. 3. Order detailed phases of gait with cards 3x, keyword for each card. 4. Closed eyes and MP done emphasising kinaesthetic perspective, say keyword for each phase. 3 series of 10 repetitions, 30 sec rest. 8 steps/repetition. 5. PP 3 series of 10 repetition, 8 steps/repetition. 6. MP in 2 imagined complex environments. 1 series of 10 repetitions, 8 steps/repetition in each environment. 7. PP in complex setting	Standard physical practice only following the same pattern as the intervention group
Schuster 2012	Group 1 (MP added): Motor task divided into 13 steps, each step imagined x5 and then practised physically x1. At end, complete task x8. Individual sessions, supervised by an instructor, task specific, same environment as physical practice, detailed and standardised instructions, internal perspective and eyes closed and no familiarisation with MI before start of intervention. Session time 45-50 min, 5 to 9 visual trials and 2 to 4 kinaesthetic trials in one session. Group 2 (MP embedded): 30 min physiotherapy, then recorded audio: 3.5 min relaxation, 14.5 min description of motor task and 2 min refocusing. Not supervised, different environments to physical practice, internal perspective and eyes closed and no familiarisation with MI before start of intervention. Session time 45-50 min. 6 to 8 visual trials and 1 to 3 kinaesthetic trials in one session. All patients kept diaries. 6 sessions in 2 weeks	Standard physiotherapy and neutral task following the same pattern as the intervention group
Seebacher 2017	30-40 min MI familiarisation in groups of 2-3. Study CD with music and verbal cueing (group 1) or metronome cues and verbal cueing (group 2). Internal perspective, kinaesthetic mode, weekly change of audio mix, home-based practice and seated position with eyes closed, self-selected time of day, 17 min practice/day, 6 days/week for 4 weeks. Record MI sessions in diary. Weekly phone calls for support and adherence	No training
Sharp 2014	Practised PP, then 30 min audio recording. 3 days/week for 8 weeks	Overground training with relaxation audio recordings
Sidaway 2005	Given instructions on kinaesthetic imagery, then performed 3 MP trials. 15 min training sessions. 3 sets of 10 repetitions, separated by 10 seconds rest, 3x/week for 4 weeks. MP group: Imagery script at start of session but did mental repetitions instead of physical repetitions. Were placed in the same environment as PP group. PP group: Physical repetitions	No training
Stenekes 2009	Active movement performed mentally following instructions to imagine initial movement, mentally hold thought in mind for 3 sec and imagine following movement. Repeat imaginary movement 10 times/session. Patients to record number of sessions performed every day. 8 sessions/day, 6 weeks	Standard post-operative rehabilitation
Timmermans 2013	6 tasks of increasing difficult. DVD guidance showing first-person perspective of task being performed, then 5 repetitions of correct performance shown with no verbal explanation and instructions to mentally practice task, then no guidance. 3x/day for 10 min, for 6 weeks. Performance assessed during intervention and if improved, DVD changed	Standard therapy and exercise therapy following the same pattern as the intervention group
Vergeer 2006	4-week programme, 30 min sessions 3x/week. Movement imagery (MI) group: Physical stretching (5-7 min warm-up, 7 stretching exercises) and imagery component (movement demonstrated, then told to imagine leg being stretched whilst simultaneously doing physical task). Stretching imagery (SI) group: Physical stretching and imagery component (told to imagine change at a cellular level previously explained with a CD, hand gestures, images and a CD)	Physical training only
Wilson 2002	Delivered by CD-ROM with software. 6 operations: Visual imagery exercises with predictive timing, relaxation, visual modelling of motor skills with video watching, mental rehearsal of skills from external perspective, mental rehearsal of skills from internal perspective and overt practice	No training
Wilson 2016	6 steps: Visual imagery exercise with predictive timing, relaxation protocol and mental preparation, mental rehearsal from external perspective, mental rehearsal from internal perspective and overt practice with mental practice between sets. 5 h individual training in 60 min sessions, 1/week for 5 weeks	No training

### Data collection and Synthesis

Data were extracted by one author using a data extraction form. The primary outcome was the efficacy of the MI training intervention, measured according to the primary outcome measure as defined by study authors. The secondary outcomes were protocol characteristics. The following items were extracted: primary outcome measure, study population, MI intervention group characteristics, control group characteristics, study primary outcome measure and rating of MI ability. The mean and standard deviation of the primary outcome measure for each study were converted to a standardised mean difference. Where post-intervention scores and follow-up scores were reported, the results of the outcome measured post-intervention only were used. Where there were several MI groups with varying length of MI practice and no data on the results of all MI groups combined, the most effective length of practice only was kept. When studies compared different types of MP, they were excluded. Where SDs were not available, they were estimated using IQR/1.35^22^, and if the IQR was not available, they were estimated based on the SDs from other studies included in the meta-analysis. The primary outcome measure used was the primary outcome stated as such by authors. Where this was not reported, the outcome measure used was the most complete measure of progress as described by the study authors, or if this was not available an outcome which reported a single measurement. Where performance was measured in different simulators (e.g. box trainer and virtual reality simulation (VRS)), the simulator which had not been used in training was used.

### Meta-analysis

Data were input into Review Manager in order to conduct meta-analysis if it satisfied the following criteria: The mean and standard deviation of the primary outcome measure were available, or could be estimated according to the methods described previously, and the study compared one group performing MI alone and one group performing MI and physical practice. Subgroup analysis was conducted based on length of training, inclusion of a relaxation component to the protocol and selection of participants based on MI ability.

### Risk of Bias

Risk of bias was assessed using the Cochrane risk of bias tool.^23^

## Results

### General Study Characteristics

**Figure 1.**
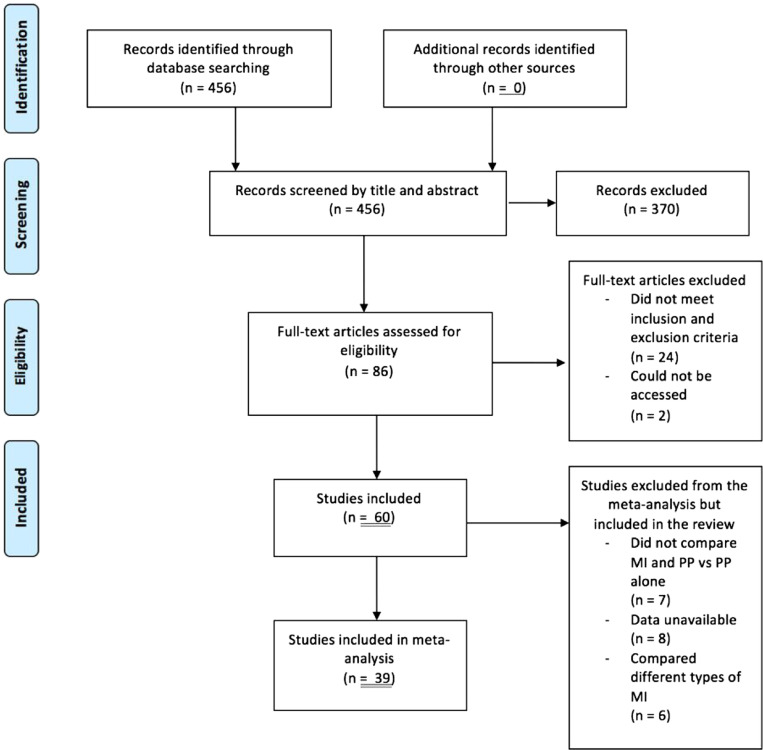
PRISMA flow diagram.

Overall, 60 RCTs were identified with a total of 2251 participants. A flow diagram of the studies’ selection process is illustrated in Figure 1. Studies were published between 1978 and 2018. Study sample sizes ranged from 10 to 112, and median was 34.5. Participants were healthy in 27 studies, had previously had a stroke in 20 studies or in 13 studies had a range of conditions including Parkinson’s disease, multiple sclerosis, arm spasticity or amputation. 6 studies only had surgical residents or trainees as participants, and 12 had healthy students.

### Primary Outcome Measures

The primary outcome was the overall effectiveness of a protocol using MI; several outcome measures were used due to the diversity of studies included. In the 12 studies^11,12,24-33^ which had medical students or trainees as their population, the primary outcome measures were the Objective Standard Assessment of Technical Skills (OSATS), variations of a Global Rating Scale, independent measures of time taken, precision and accuracy in completion of task or a purpose-built checklist. The remaining studies used task-specific measures such as the Fugl-Meyer assessment for stroke^50,56,61^ or broader measures of function such as using a goniometer for range of motion^40-43^.

### Outcomes

25 (42%) studies found that the intervention group using MI did not perform better than the control group.^6,11,24,26-31,34-49^

In 35 studies (58%), the intervention group performed better than the control group.^3,12,25,32,33,50-79^ In 29/35 studies (83%), the intervention group which did MP and standard physical practice (rehabilitation, physiotherapy and surgical training) performed better than the control group which did only standard physical practice.^3,12,25,32,33,50-70,76,78,79^ There was no trend found between these study results and the use of MI ability assessment, the outcome measures used or the length of interventions.

A summary of all data extracted is presented in Tables 1 and 2.

### Secondary Outcomes: Intervention Duration and Number of Motor Imagery Sessions

The median duration of each individual MI session was 27.5 minutes (range <1 minute-120 minutes).

In studies where the intervention group performed better than control, the median was 30 minutes (range <1 minute-120 minutes). The median number of MI sessions completed was 11 (range 1-1680); and 13 (range 1-1680) in studies where the intervention group performed better than control. The median duration of intervention across all studies was 22.4 days (mode 1, range 1-70), and in studies where the intervention group performed better than control, it was 24 days (mode 42, range 1-70).

### Secondary Outcomes: Intervention Content

In 22 studies,^11,12,24,25,27,29,32,41,42,44,47,48,50,51,53,58,59,61,65,66,69,77^ the MI sessions began with a brief period of relaxation lasting <5 minutes. Out of these, 13 of them found the intervention group performed better than control.^12,25,32,50,51,53,58,59,61,65,66,69,77^ In 7 studies, there was explicit mention of the use of sensory cues for visualisation, giving an indication of the specificity of the instructions given to participants.^3,31,39,51,59,65,69^ The level of detail to which the protocols were reported was not consistent across the studies reviewed. In 13 studies, the MI sessions included several repetitions of MI with periods of rest in between.^40,42,43,51,53,55,57,58,60-62,68,75^ Out of these, 10 found the intervention performed better than control.^51,53,55,57,58,60-62,68,75^ 7 studies mention a refocusing period at the end of the MI session.^42,51,59,61,65,69^

### Secondary Outcomes: Motor Imagery Ability Assessment

Certain studies measured participant ability to conduct MI, as MI ability differs in a healthy population,^80^ and can be measured using validated imagery questionnaires, such as the Mental Imagery Questionnaire (MIQ), the Mental Imagery Questionnaire Revised, Second Edition (MIQ-RS) or the Vividness of Mental Imagery Questionnaire (VMIQ).^81^ In patients who have a neurological impairment, the Kinaesthetic and Visual Imagery Questionnaire (KVIQ)^82^ can be used, as can mental chronometry, which has been shown to correlate with MI ability in healthy and non-healthy patients. 23 studies (38%) used the MIQ, MIQ-RS, VMIQ, VMIQ-2, VVIQ, mental chronometry, KVIQ or time-dependent motor imagery (TDMI) to select participants based on MI ability.^6,12,24,34,38,40-43,45,47,48,53,55,57,60-62,64,66,68,72,79^ Out of these, 12 (52%) reported better outcomes in the intervention group compared to control.^12,53,55,57,60-62,64,66,68,72,79^

### Meta-analysis

**Figure 2.**
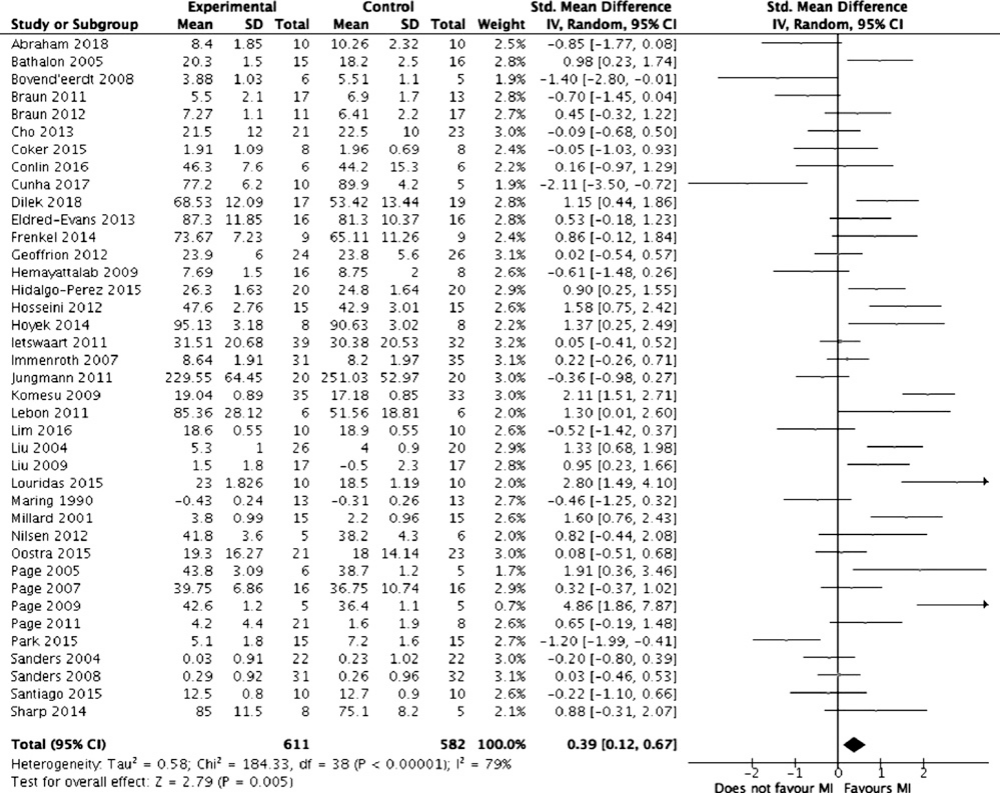
Forest plot comparing mental training interventions to control.

**Figure 3.**
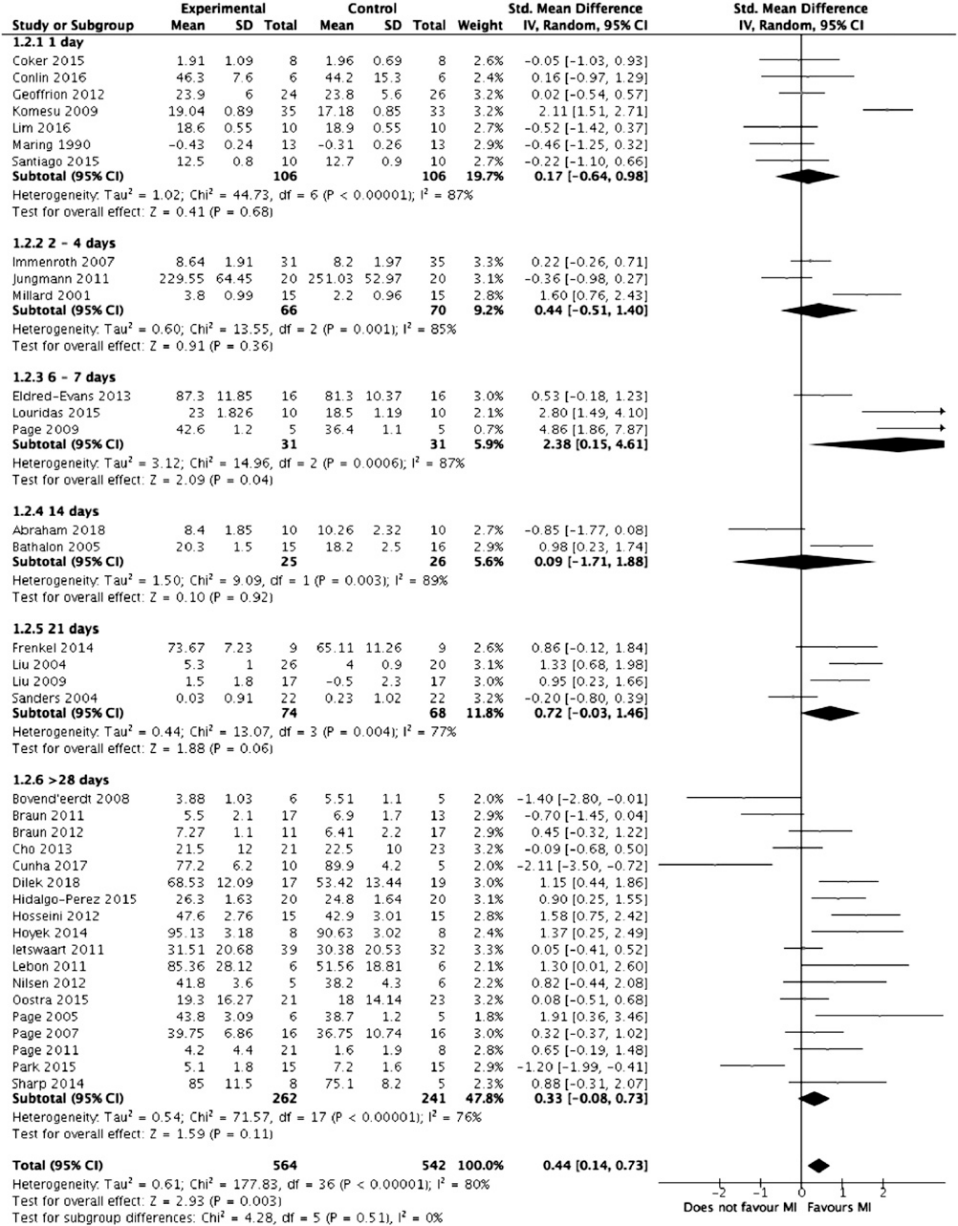
Forest plot comparing mental training interventions of 1 day, 2-4 days, 6-7 days, 14 days, 21 days and >28 days duration.

**Figure 4.**
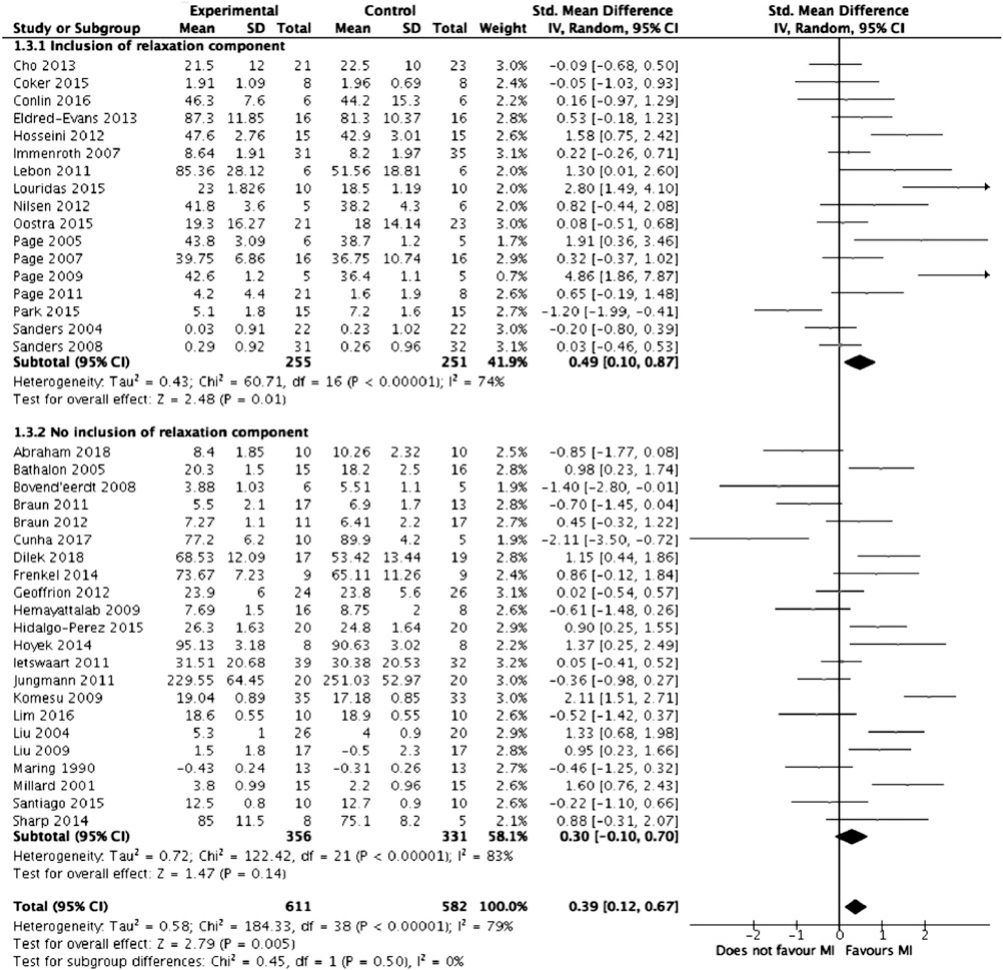
Forest plot comparing mental training interventions with a relaxation component to mental training interventions with no relaxation component.

**Figure 5.**
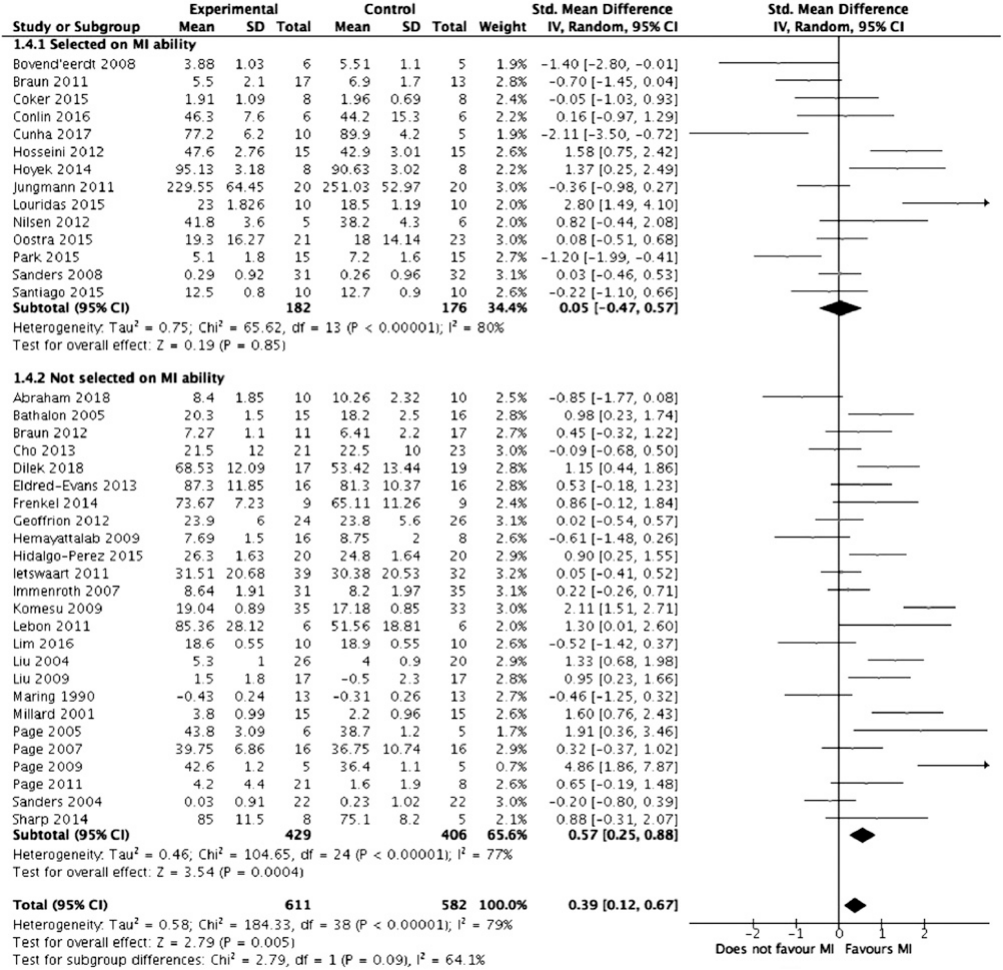
Forest plot comparing studies where participants were selected based on mental training ability to studies where participants were not.

Meta-analysis was performed on the 39 studies eligible for inclusion. Figure 2 summarises the results of the meta-analysis. Overall, mental imagery was associated with improved outcomes (z = 2.79, *P* = .005) but with high heterogeneity between the studies (I^2^ = 79%, *P* < .00001). Figures 3-5, respectively, detail the results of subgroup analyses based on length of training, relaxation and selection of participants based on MI ability.

### Risk of Bias Assessment

The risk of ‘other’ bias was classed as high in 26 studies, primarily due to selecting participants based on MI ability. The risk of selective reporting bias was unclear for the majority of studies as only few had previously published a protocol which could be referred to. 7 studies had a risk of bias which was classed as low for 5 or more types of bias and unclear for 2 or less types of bias, which the authors consider to be an overall low risk of bias. Of these, 2^12,55^ found the intervention group performed better than control on outcomes measures and 5^24,28,42,43,46^ found they performed the same or worse. A summary of the risk of bias is presented in Table 3.

**Table 3.** Risk of Bias.

Study	Random Sequence Generation	Allocation Concealment	Blinding of Participants and Personnel	Blinding of Outcome Assessment	Incomplete Outcome Data	Selective Reporting	Other Sources of Bias - MI Ability Assessment
Abraham 2018	Low	Unclear	Unclear	Unclear	Low	Unclear	Unclear
Asa 2014	Unclear	Unclear	Unclear	High	Low	Unclear	High
Bathalon 2002	Unclear	Unclear	Low	Low	Low	Unclear	High
Bovend'Eerdt 2008	Low	Unclear	High	Low	Low	Unclear	Low
Braun 2011	Unclear	Unclear	Unclear	Low	Low	Unclear	Low
Braun 2012	Low	Unclear	High	Low	Low	Low	High
Cho 2013	Low	Low	Unclear	Unclear	Low	Unclear	High
Coker 2015	Unclear	Unclear	Unclear	Unclear	Low	Unclear	Low
Conlin 2016	Low	Unclear	Unclear	Low	Low	Low	Low
Cunha 2017	Unclear	Unclear	Unclear	Unclear	High	Unclear	High
Dilek 2018	Low	Low	Unclear	Low	Low	Unclear	High
Eldred-Evans 2013	Low	Low	Unclear	Low	Unclear	Unclear	High
Frenkel 2014	High	High	Low	High	Low	High	Unclear
Geoffrion 2012	Low	Low	Unclear	Low	Low	Unclear	High
Gomes 2014	Unclear	Unclear	Unclear	Low	Low	Unclear	High
Guillot 2009	Unclear	Unclear	Unclear	Unclear	Low	High	Low
Hemayattalab 2009	Unclear	Unclear	Unclear	Unclear	Low	Unclear	High
Hidalgo-Perez 2015	Low	Low	High	Low	Low	Unclear	High
Hosseini 2012	Unclear	Unclear	Unclear	Low	Low	Low	Low
Hoyek 2014	Unclear	Unclear	High	High	Low	Unclear	Low
Ietswaart 2011	Low	Unclear	Low	Low	Low	Unclear	Low
Immenroth 2007	Low	Low	High	Unclear	Low	Unclear	High
Jungmann 2011	Unclear	Unclear	Unclear	Unclear	Low	Unclear	Low
Kim 2013	Unclear	Low	Unclear	Unclear	Low	Unclear	High
Kim 2018	Low	Unclear	High	High	Low	Low	Low
Komesu 2009	Low	Low	Unclear	Low	Low	Unclear	High
Lebon 2011	Unclear	Unclear	Low	Low	Low	Unclear	High
Lim 2016	Low	Unclear	Low	Low	Low	Unclear	Low
Liu 2004	Unclear	Low	Unclear	Low	Low	Unclear	High
Liu 2008	Unclear	Unclear	Unclear	Low	Low	Unclear	High
Liu 2009	Low	Low	Unclear	Low	Low	Unclear	High
Losana-Ferrer 2018	Low	Low	Unclear	Low	Low	Unclear	Low
Louridas 2015	Low	Low	Low	Low	Low	Low	Low
Malouin 2009	Low	Unclear	Unclear	Low	Low	Unclear	Low
Maring 1990	Unclear	Unclear	Unclear	Unclear	Low	High	High
Mendoza 1978	Unclear	Unclear	Low	Low	Low	Unclear	High
Millard 2001	Unclear	Unclear	Unclear	Low	Low	Unclear	High
Mulla 2012	Low	High	Unclear	Unclear	Low	Unclear	High
Nicholson 2018	Low	Low	High	Low	Low	Unclear	Low
Nilsen 2012	Unclear	Unclear	Low	Low	Low	Unclear	Unclear
Oostra 2004	Low	Unclear	Unclear	Low	Low	Unclear	Low
Page 2005	Low	Unclear	High	Low	Low	Low	High
Page 2007	Low	Unclear	Low	Unclear	Low	Unclear	High
Page 2009	Low	Unclear	Unclear	Unclear	Low	Unclear	High
Page 2011	Low	High	Unclear	Low	Low	Low	High
Park 2015	Low	Unclear	Unclear	Low	Low	Unclear	Low
Sanders 2004	Unclear	Low	Low	Low	Unclear	Unclear	Low
Santiago 2015	Low	Low	Unclear	Low	Low	Unclear	Low
Schuster 2012	Low	Low	Low	Low	Low	Low	Low
Seebacher 2017	Low	Low	High	Unclear	Low	Low	High
Sharp 2014	Low	Low	Unclear	Low	Unclear	Unclear	High
Sidaway 2005	Unclear	Unclear	Unclear	Unclear	Low	Unclear	High
Stenekes 2009	Unclear	High	Unclear	Unclear	Low	Unclear	Low
Timmermans 2013	Low	Low	High	Low	Low	Low	Low
Vergeer 2006	Unclear	Unclear	Unclear	Low	Low	Low	Low
Wilson 2002	Low	Unclear	Unclear	Low	Low	Unclear	High
Wilson 2016	Unclear	Unclear	High	Low	Unclear	Unclear	High

## Discussion

This review assessed only RCTs evaluating the effectiveness of various MI protocols across the fields of sports, neurorehabilitation, education and medical education. The aim was to extract the components of a successful MI protocol. The authors hypothesised these components might be universal to MI training applied to several different indications, hence the inclusion of a heterogeneous sample of studies. In addition, MI programmes for surgical training remain novel, with few studies having specifically evaluating its effectiveness on surgeons. Broadening the search across several disciplines allowed protocol components never included in surgical training programmes to be considered.

Performing MI in addition to standard rehabilitation or training led to improvements in the majority of trials (83%).This is consistent with the concept that MI is a valuable tool when added to existing training. Based on current understanding of the neurological processes of MI, it can be speculated that protocols which demonstrate improvement in non-surgical fields can be extrapolated to surgical training, due to the fact all are focussed on motor skill learning. This could be particularly true for healthy populations improving on a specific skill - such as athletes. Surgical trainees and athletes have in common a healthy physical baseline and the goal of improving a specific motor skill. However, the authors acknowledge the methodological limitation of assuming similarities between populations. Overall, there were very few studies which specifically tested MI skills in surgical residents; this is a novel method of training in this field which must be tested further. This method could be used to improve a range of motor skills, ranging from generic surgical skills to patient-specific skills. Motor imagery-based training could be a supplement to standard surgical training.^10,31^

Studies where the intervention group performed better than control on outcomes had a median duration of intervention of 30 minutes, with a median of 15 MI sessions completed in 26 days. This is equivalent to performing MI more than once every 2 days. An online surgical training course, where trainees conducted a short amount of imagery, regularly and at their convenience, would fit these requirements. Indeed, there were 7^12,27,30,52,55,60,74^ studies in which subjects were instructed to perform MI independently at home and record their progress. In the study by Louridas et al,^12^ surgical trainees were given 7 days to perform MI at home and had follow-up calls and feedback. Only 2 of these 7 studies, by Jungmann et al^30^ and Mulla et al,^27^ did not see an improvement in the intervention group compared to control. They were also the only 2/7 studies which used medical students as their population. This means a MI training protocol for surgical education could be in a format which allowed subjects to access training in their own time.

Regarding the content of MI interventions, the level of detail provided across the studies review varied widely, making direct comparisons of protocols and associated outcomes difficult. However, the following elements could be incorporated into the structure of MI protocols in the interest of standardising their format and enabling direct comparison of outcomes in future research: a period of relaxation <5 minutes long prior to starting MI proper; detailed instructions involving specific sensory cues, a predetermined number of sets of repetitions of MI to be performed in each session and a refocusing period to close the MI session.

Given that there was no association between MI ability and technical performance (when compared to control), this indicates that baseline MI ability may not be an important factor for a MI training programme.

Given the heterogeneity of study outcomes measured and the variability of populations studied, no extrapolation can be made of the primary outcome most suitable for measuring the effectiveness of an MI training protocol. Relevant to surgical education MI training, a variety of primary outcome measures were used amongst the medical student and resident populations. These were variations of a pre-established checklist and objective measurements such as time and accuracy.

In 9 of the studies^24,27,29,30,34-36,39,40^ where the intervention group performed worse or equivalent to the control group, subjects were students or healthy participants for whom the benefit of the study was not obvious: they did not have an intrinsic motivation to perform well on the outcomes measured such as increased function of a limb following a stroke or improved surgical technique. This may indicate that for MI interventions to be successful, participants need to be self-motivated, and in the context of surgical education, surgeons should only undergo MI training if they see potential benefit in it. However, this is difficult to establish in the heterogeneous group of studies reviewed here and would benefit from further research focussed on surgical trainees’ motivation to use MI with their performance after training. Guillot’s article did explore the relationship between intrinsic motivation and MI in the opposite direction and suggests that MI does enhance intrinsic motivation.^16^

A number of limitations to these results need to be considered. The majority of the studies were intrinsically biased as the subjects who received the intervention could not be blinded. Another limitation is the heterogeneity of studies included in this review. Studies included represented many applications of MI training, which may limit the generalisability of findings. Only 12 studies focussed on the application of MI training directly to surgical trainees or medical students. Further research is required to demonstrate that the findings from this review can be translated to surgical education. Furthermore, variations in study methodologies limited pooled analysis.

Following this review, more research focussing on the implementation of MI training protocols in surgical education is needed, in addition to the acceptability of such training measures among trainees and surgeons. The results of this review may aid in constructing a purpose-built MI training programme to evaluate its efficacy on surgical trainees specifically.

## Conclusions

This comprehensive systematic review and meta-analysis has identified several characteristics linked to successful MI training in sports or neurorehabilitation that can be used to construct MI training protocols for use in surgical education. It must be highlighted that this review and analysis included a wide range of studies in different fields. However, certain components found to be linked to successful programmes could be extrapolated to surgical training, based on current understanding of neurological processes of MI. A successful MI training programme could be delivered in parallel to existing surgical training, in a flexible format allowing surgeons to undertake several MI sessions in a self-directed manner. A single MI session conducted by a senior surgeon could include a brief period of relaxation, followed by several sets of repetitions of MI, and a refocusing period. Providing guidance on the construction of effective MI training protocols will allow replicability of trials investigating the best way to deliver MI training. This is a step towards the development of a surgical MI training programme, as a low-cost, low-risk tool to enhance practical skills. Further research will be required to evaluate the use of MI in a purpose-built surgical training programme.

## Appendix A

**Table:**

Detailed Search Strategy
PubMed
((‘Motor imagery’) OR (‘mental imagery’) OR (‘mental practice’) OR (‘mental training’)) AND ((‘randomised controlled study’) OR (‘randomised controlled trial’))
265 results
Ovid (PsycINFO, Embase and MEDLINE)
((Motor imagery) OR (mental imagery) OR (mental practice) OR (mental training)) NOT (computerised OR computer)
Filter: Randomised controlled trial
191 results

## Appendix B. List of Abbreviations Used in Tables 1 and 2.

**Table:**

ACL	Anterior cruciate ligament
AMIT	Advanced Mechanical Technology Inc
BT	Box training
CMIT	Constraint-induced movement therapy
EMG	Electromyography
FAT	Frenchay arm test
FM	Fugl-Meyer assessment test
GRS	Global Rating Scale
IST	Intelligence Structure Test
IVI	Internal visual imagery
KG	Kinesiology
KIN	Kinaesthetic imagery
KVIQ-20	Kinaesthetic and visual imagery questionnaire 20
LBT	Line bisection test
MABC	Movement Assessment Battery for children
MCTE	Motor control therapeutic exercise
MI	Mental imagery, motor imagery
MIQ-RS	Motor Imagery Questionnaire-Revised
MMSE	Mini-mental state examination
MP	Mental practice
n/a	Not available
OSATS	Objective Standard Assessment of Technical Skills
OT	Occupational therapy
PP	Physical practice
SCT	Star cancellation test
SI	Stretching imagery
TDMI	Time-dependent motor imagery
VMIQ-2	Vividness of Movement Imagery Questionnaire 2
VR	Virtual reality
VRS	Virtual reality simulation
